# Sex-specific population structure, natural selection, and linkage disequilibrium in a wild bird population as revealed by genome-wide microsatellite analyses

**DOI:** 10.1186/1471-2148-10-66

**Published:** 2010-03-08

**Authors:** Meng-Hua Li, Juha Merilä

**Affiliations:** 1 Ecological Genetics Research Unit, Department of Biosciences, PO Box 65, FI-00014 University of Helsinki, Finland

## Abstract

**Background:**

Sexual dimorphism in ecologically important traits is widespread, yet the differences in the genomic architecture between the two sexes are largely unexplored. We employed a genome-wide multilocus approach to examine the sexual differences in population subdivision, natural selection and linkage disequilibrium (LD) in a wild Siberian jay (*Perisoreus infaustus*) population, using genotypes at a total of 107 autosomal and Z-chromosomal microsatellites.

**Results:**

Mean observed heterozygosity was significantly higher in females (*H*_O _= 0.567) than in males (*H*_O _= 0.532), and autosomal markers (*H*_O _= 0.561) were more variable than Z-chromosomal markers (*H*_O _= 0.512). Genetic differentiation (*F*_ST _= 0.002, *P *< 0.05) between the two sexes was low but significant and males were on average significantly more genetically related to each other than females. Genomescan analyses revealed that 3 out of 101 (3%) autosomal loci were under directional selection, while 4 out of 6 (67%) Z-chromosomal markers were indicated to be under balancing selection. This suggests a significantly greater but contrasting selection force on the Z-chromosome in comparison to autosomes, which is consistent with an overall significantly (*P *< 0.05) lower *F*_ST_value for Z-chromosomal (-0.014, 95% CI: -0.025 - -0.011) than for the autosomal loci (0.003, 95% CI: 0.001 - 0.004). Analysis of syntenic marker pairs revealed high levels of LD in both sexes but significantly (*P *< 0.05) lower levels of LD in the females both on autosomes and Z-chromosome, probably due to the higher rate of dispersal and the higher recombination rates on autosomes, as well as the pseudoautosomal markers. In both sexes LD decayed rapidly with genetic distance in a similar fashion on autosomes, while a more rapid decay of LD in Z-chromosome was detected in females than in males.

**Conclusion:**

We conclude that there are many clear differences in genomic architecture between the sexes studied here which can be at least partly understood in the light of higher dispersal rate of females as compared to males and the unusual structure of the Z-chromosome of the species.

## Background

Large differences in the forces of evolution - mutation, recombination, selection, gene flow, and genetic drift - are known to occur between males and females (e.g. [1]). Therefore, understanding the relative importance of evolutionary forces that shape patterns of sex-specific genomic dimorphism is essential to our understanding of the genetic basis of sexual dimorphism and sex-specific gene expression (e.g. [2,3]). Recently, growing efforts have been invested on elucidating the fine scale genetic architecture of sexual dimorphism in complex phenotypes (e.g. [3,4]). However, little is known about the genetic architecture underlying sex-biased evolutionary processes at the genomic level (but see [1,5]), especially in the wild.

In most birds sexual differences in behaviour with respect to mating and dispersal practices are a rule rather than exception (see the reviews in [6-8]). Likewise, sex-specific selection for example in the form of competitive exclusion of individuals of one sex during recruitment or establishment phases is a commonplace occurrence in birds (e.g. [9]), as is also sexual dimorphism in size (see e.g. [10]) and plumage traits [11]. As a consequence, also sexual dimorphism in genomic architecture - including the extent of linkage disequilibrium (LD) - is expected to occur both for selective and demographic reasons. While detailed understanding of the extent and patterns of LD in the two sexes is interesting in itself, this knowledge will also facilitate the choice of appropriate methodology for sex-specific QTL mapping in the wild. In addition, inbreeding avoidance, sexual conflict, and sex-biased investment at the population level can cause non-random mating resulting in differences in genotypic distribution between the sexes (e.g. [12,13]). To this end, a multilocus approach as used e.g. in *Drosophila*, maize and humans (e.g. [14,15]) is a powerful way to disentangle the effects of sex-related evolutionary forces on genomic variation.

The Siberian jay (*Perisoreus infaustus*) is a passerine bird which has been subject to considerable ecological and evolutionary research during the past decades. It exhibits sexual dimorphism in morphological measurements [16], nepotistic behaviour [17], genetic structuring [18], lifetime reproductive success and dispersal patterns ([19], Gienapp and Merilä, unpublished results). Moreover, sex-specific genome-wide heterogeneity in recombination rates and linkage patterns for both autosomes and the Z-chromosome have been recently detected [20].

Here, we adopted a genome-wide multilocus approach to examine the sex-specific genomic differences in Siberian jays taking advantage of a total of 107 microsatellite markers genotyped in 172 males and 177 females. In particular, we analysed and compared Z chromosomal and autosomal microsatellites to identify possible sex-specific processes that may have shaped genomic patterns of variability. We also investigated the possible sexual dimorphism in magnitude and extent of LD. In particular, using sex-specific linkage maps, we investigated the decay of LD with genetic distance in the sexes. The main objectives were to: 1) determine the pattern of variability and genetic differentiation between sexes in both autosomal and Z-chromosomal loci; 2) investigate the potential signatures of natural selection related to sex on autosomal and Z-chromosomal markers; and 3) investigate the differences in extent and pattern of LD between the sexes.

## Results

### Genetic diversity, differentiation and relatedness

**Figure 1:**
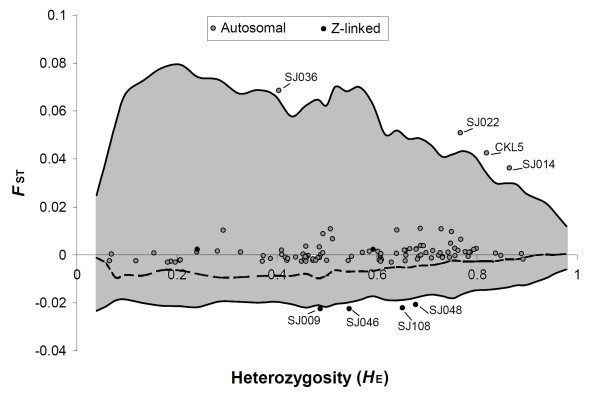
**Distribution of observed *F*_ST _values for each loci as a function of their Nei's unbiased heterozygosity (*H*_E_)**. The simulated median line and 95% confidence limits are represented by dashed and solid lines, respectively, for the Fdist2 method. Gray shading indicates area on the graph within the 95% confidence limits.

Locus specific heterozygosities and degree of genetic differentiation are shown in Figure 1 and Table 1. A significantly lower average observed heterozygosity was found in males (*H*_O _= 0.532 ± 0.004) than in females (0.567 ± 0.004; paired *t*-test: *t*_212 _= 1.16, *P *< 0.01; Table 1). The mean unbiased estimated heterozygosity for autosomal markers (*H*_E _= 0.566 ± 0.003) was significantly (unpaired t-test: *t*_105 _= 3.36, *P *< 0.05) higher than that for Z-chromosomal markers (*H*_E _= 0.512 ± 0.001; Figure 1). *F*_ST _values for individual loci among sexes varied from -0.023 (SD = 0.011) at SJ046 on Z-chromosome to 0.069 (SD = 0.037) at SJ036 on autosome. Overall genetic differentiation across all the loci was *F*_ST _= 0.002 (95% CI 0.001 - 0.003, *P *< 0.05) between males and females, with *F*_ST _= 0.003 (95% CI 0.001 - 0.004, *P *< 0.05) for autsosomal markers and *F*_ST _= -0.014 (95% CI -0.025 - -0.011, *P *< 0.05) for Z-chromosomal markers. Across all the loci, the mean *F*_IS _was 0.035 (± 0.014) over all the samples and deviated significantly (*P *< 0.05) from zero, while being 0.053 (± 0.027, *P *< 0.05) for males and -0.018 (± 0.013, *P *< 0.05) for females. The average pairwise relatedness was 0.036 (SE: 0.006, 95% CI: 0.032 - 0.039) for males, 0.019 (SE: 0.003, 95% CI: 0.017 - 0.022) for females and 0.041 (SE: 0.008, 95% CI: 0.038 - 0.045) for males and females combined (Table 1).

**Table 1:** Summary statistics of observed heterozygosity (*H*_O_), Nei's unbiased heterozygosity (*H*_E_), *F*-statistics (*F*_IS _and *F*_ST_), and relatedness (*R*) averaged for males, females and the overall sample at autosomal and Z-chromosomal microsatellites in the Siberian jays.

					*F*_ST _(95% CI)	
					
Sex	*H*_O_ (± SD)	*H*_E_ (± SD)	*F*_IS_ (± SD)	*R* (± SE, 95% C.I.)	Autosomal	Z-chromosomal
Male	0.532 (0.004)	0.559 (0.019)	0.053* (0.027)	0.036 (0.006, 0.032 - 0.039)	-	-
Female	0.567 (0.004)	0.562 (0.019)	-0.018* (0.013)	0.019 (0.003, 0.017 - 0.022)	-	-

Overall	0.550 (0.003)	0.561 (0.019)	0.035* (0.014)	0.041 (0.008, 0.038 - 0.045)	0.003* (0.001 - 0.004)	-0.014* (-0.025 - -0.011)
					
					0.002 (0.001 - 0.003)*	

* *P *< 0.05

### Evidence for selection between males and females

The FDIST2 analyses identified eight loci as outliers showing footprints of natural selection between males and females at the 0.5% significance level (Figure 1; Table 2). Of the eight significant loci, four were autosomal (SJ014, SJ022, SJ036 and CKL5) with higher than expected *F*_ST_values indicating directional selection, while four Z-chromosomal loci (SJ009, SJ046, SJ048 and SJ108) appearing in the lower tail of the *F*_ST _distribution showed signatures typical of balancing selection (Figure 1).

**Table 2:** Summary statistics of outliers detected between male *vs*. female Siberian jays, using FDIST2 and BAYESFST methods.

Locus	Location^*a*^	FDIST2				BAYESFST		
			
		*F*_ST_	*H*_E_	*P*	Selection	*F*_ST_	*P*	Selection
SJ014	A-LG4	0.036	0.865	0.0032*	directional	0.019	0.08	-
SJ022*	A-LG1	0.051	0.766	0.0022*	directional	0.024	0.03*	directional
SJ036*	A-LG2	0.069	0.404	0.0034*	directional	0.038	0.01*	directional
CKL5*	A-LG2	0.043	0.819	0.0015*	directional	0.023	0.03*	directional
SJ009*	Z	-0.023	0.467	0.0027*	balancing	0.006	0.01*	balancing
SJ046*	Z	-0.023	0.544	0.0041*	balancing	0.005	0.03*	balancing
SJ048*	Z	-0.021	0.677	0.0033*	balancing	0.005	0.04*	balancing
SJ108*	Z	-0.022	0.651	0.0029*	balancing	0.005	0.04*	balancing

^*a *^A, autosome; Z, Z-chromosome; LG, linkage group

* Significant value of 5% level for the BAYESFST method and 0.5% level for the FDIST2 method, and locus labels marked with asterisks if both methods indicate a significant result

Bayesian *F*_ST_-test based on a hierarchical regression model indicated three autosomal loci (SJ022, SJ036 and CKL5) as directionally selected and four Z-chromosomal loci (SJ009, SJ046, SJ048 and SJ108) to be under balancing selection (Table 2). Thus, seven of the loci (SJ009, SJ022, SJ036, CKL5, SJ046, SJ048 and SJ108) were picked up by both methods giving support to their status as outliers due to selection. In addition, locus SJ014 can be seen as candidate affected by directional selection, but only one of the two tests supported this statistically.

**Figure 2:**
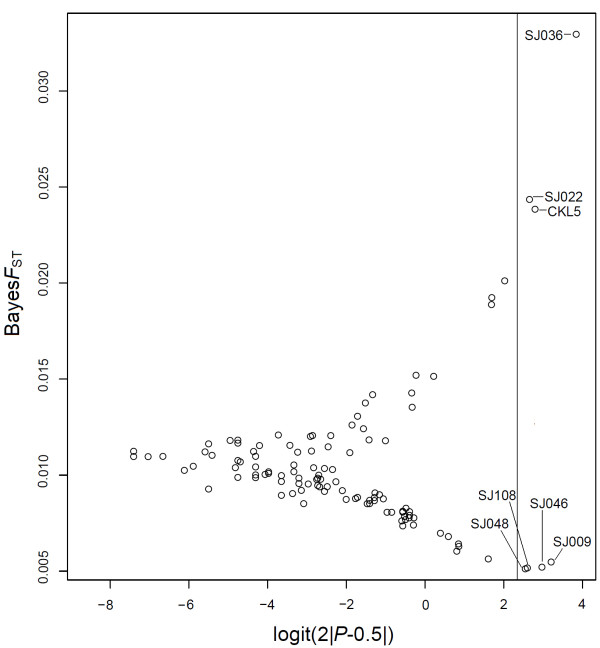
**Results of the Bayesian *F*_ST_-test**. The solid line indicates the critical cut-off point for the *P*-value at the 5% level.

Out of the 107 microsatellite analysed, the four Z-chromosomal loci (SJ046, SJ048, SJ009, and SJ108) under balancing selection showed the lowest distribution of *F*_ST _values in both tests being as negative outliers in the BAYESFST results; whereas the other four autosomal loci under directing selection detected by one or both of the tests show the highest differentiation values in both analyses (Figures 1 and 2).

### Patterns of LD

**Figure 3:**
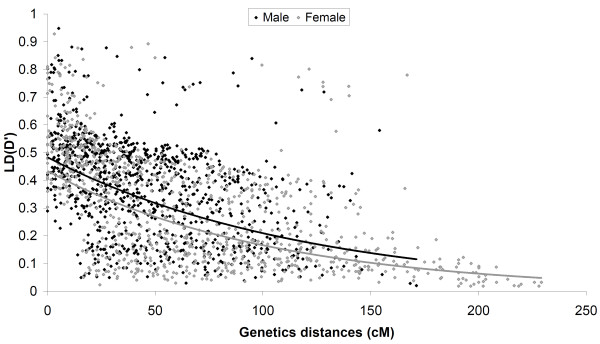
**Linkage disequilibrium as measured by *D*' as a function of genetic distances (cM) between autosomal syntenic markers**. The dark and grey lines give exponential trend line fits for males and females, respectively.

Figure 3 and Table 3 show the distribution of *D*' values for the autosomal syntenic marker pairs as a function of genetic distance (in cM) in both females and males. In both cases, *D*' decayed with distance varying between 0.02 - 0.947 in males and between 0.018 - 0.928 in females (see Figure 3). In total, significantly lower mean *D*'-value for the autosomal markers was revealed in females than in males (Males: 0.378 ± 0.145; Females: 0.299 ± 0.126; unpaired *t*-test: *t*_1874 _= 9.13, *P *< 0.001). Mean *D*'-values were 0.574 (± 0.162) in males and 0.532 (± 0.154) in females for markers separated by 10 cM or less, while at the distance intervals of > 10 cM between marker pairs are all <0.5 in both sexes (Table 3). All the mean *D*'-values are lower than that in the sample of pooled sexes (see [21]). Thus, the 'half-length' of LD (measured as the distance at which mean *D*' falls to 0.5) is *ca*. 10 cM, half of that measured in the pooled sample of males and females (*ca*. 20 cM; see [21]). For the autosomal marker pairs, the mean *D*'-value decreased with increasing genetic distance and was systematically lower in the females than in the males at comparable distances (Figure 3). Both of the negative correlations between LD and genetic distance were statistically significant (*F*-test, *P *< 0.01). The coefficient of determination (*R*^2^) was 0.219 for males and somewhat higher (*R*^2 ^= 0.367) for the females when exponential trend lines were fitted to the data. As further indicated in Figure 3, *D*' decays relatively rapidly in the first tens of centimorgans, while for marker pairs spaced by > *ca*. 100 cM slower decreases are detected. However, significant (*P *< 0.05) and strong associations (*D*' > 0.5) were observed also for a few comparisons among loci separated by > 100 cM in both sexes (Figure 3).

**Table 3:** LD summary statistics of mean values of the Lewontin's normalised *D*' (Lewontin 1964) in all linkage groups (autosome and Z-chromosome) separately for syntenic and nonsyntenic markers in males and females.

	Syntenic (cM)								
	
Sex	Autosome							**Z-Chr**.	Nonsyntenic
		
	0-10	10-20	20-40	40-60	60-100	100-171.1	171.1-229.2	0-48.9	
Males	0.574 (0.162)*	0.495 (0.151)	0.389 (0.142)	0.354 (0.131)	0.299 (0.124)	0.234 (0.110)	-	0.517 (0.176)	0.153 (0.063)
Females	0.532 (0.154)	0.443 (0.151)	0.345 (0.129)	0.261 (0.122)	0.232 (0.117)	0.196 (0.104)	0.061 (0.034)	0.469 (0.169)	0.104 (0.034)

* Value in parenthesis are standard errors for *D*'.

**Figure 4:**
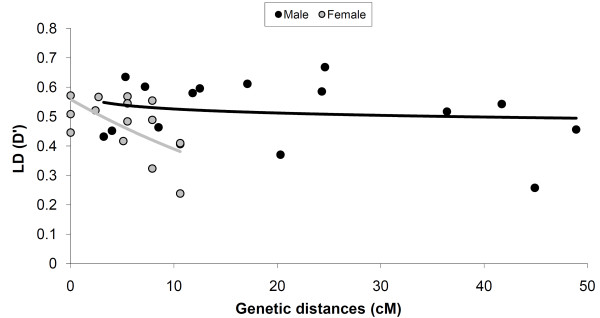
**Linkage disequilibrium as measured by *D*' as a function of genetic distances (cM) between Z-chromosomal syntenic markers**. The dark and grey lines give exponential trend line fits in males and females, respectively.

**Figure 5:**
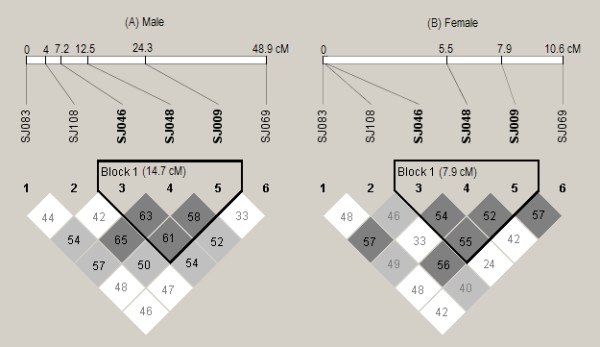
**Detailed view of the extent and significance of LD in males and females using Haploview 4.0 program**. The LD blocks defined are as described in the Materials and Methods section. Numbers in the blocks indicate the percentage of the LD metric *D*' values. Shadings indicate Fisher's exact test significance levels: white, *P *> 0.05; light shading, *P *< 0.05; dark shading, *P *< 0.01. The lengths of male- and female-specific linkage groups in the figures are not in proportion.

We also compared the level of pairwise LD measured for Z-linked markers as a function of genetic distance in males and females. The estimates of *D*'-values are significantly (unpaired Student's *t*-test: *t*_28 _= 1.25, *P *< 0.001) lower in females (mean *D*' = 0.469) than in males (mean *D*' = 0.517; Table 3). In both sexes, the *D*' decayed as a function of genetic distance. However, at the genetic distances ≤ 10.6 cM (the length of female-specific Z-chromosome linkage group) a more rapid decay of *D*' was detected in females than in males (Figure 4). When logarithmic trend lines were fitted to the data of females and males, the coefficients of determination (*R*^2^) were 0.319 and 0.025, respectively. We also detected significant (*P *< 0.01) LD with *D*' > 0.5 at all locus pairs among three adjacent loci (SJ046, SJ048, and SJ009), *i.e*. LD block, in both males and females (Figure 5).

Gametic LD was also determined for the 4718 nonsyntenic marker pairs in both females and males separately. For the nonsyntenic marker pairs *D*' varies between 0.005 - 0.431 in males and 0.005 - 0.373 in females (data not shown). The mean *D*'-values were 0.153 (± 0.063) in males and 0.104 (± 0.034) in females (Table 3), significantly lower than that for the syntenic marker pairs (Males: 0.378 ± 0.145, *t*_5669 _= 5.11, *P *< 0.0001; Females: 0.299 ± 0.126, *t*_5669 _= 6.24, *P *< 0.0001).

## Discussion

### Levels and patterns of genetic diversity, sexual differentiation and relatedness

Both sexes of Siberian jays exhibited substantial levels of genetic variation. More interestingly, males were significantly less heterozygous than females. This genome-wide pattern appears compatible with the female-biased dispersal in this species (see below). Also differential natural selection on the two sexes could contribute to this observation. For instance, more intensive within-sex competition could reduce genetic variability in males more than in females (see [8]). Our findings of lower levels of diversity in males and on the Z-chromosome relative to the autosomes are concordant with results of previous studies based on nucleotide sequences or microsatellite variation in a wide range of species including the collared flycatcher [22], chicken [23], human [24,25], house mice [26] and *Drosophila *[27,28]. Indeed, many evolutionary models, such as recent population bottleneck and recurrent selective sweeps are expected to reduce the levels of Z-linked (or X-linked) genetic diversity relative to autosomal variability (see [1]).

We found evidence for a significant sex-specific pattern of population genetic structure at autosomal microsatellites. The observed genetic differentiation between males and females was unexpected given that males and females are from the same cohorts and share genealogies within the pedigrees: their autosomal markers are mixed at each generation. Therefore, long-term evolutionary forces such as genetic drift or mutation could not generate autosomal genetic difference between sexes [29]. Moreover, selective effects were rarely (4/101, 3.97%) detected in the autosomal microsatellites. Thus, the short-term (one generation) effect of migration, for example the differential patterns of dispersal between sexes, appear to provide the most likely explanation. Complete data on life-time reproductive success and dispersal history for a large number of males and females collected for over 30 years have revealed that natal dispersal distance was related to sex and dispersal-timing: females and early dispersers travelled on average farther than males and delayed dispersers (Gienapp and Merilä, unpublished results). Our genetic data also corroborate these findings, with a tendency for negative *F*_IS _and lower genetic relatedness among females. The significant positive *F*_IS _observed in males is consistent with this argument as well. Alternative explanations for the sex-specific genetic differentiation would be differential viability, lifespan or hatching rate between sexes, which can lead to the allele frequency difference between females and males detected by *F*_ST _(see [8,29]). Unfortunately, given the available data in the Siberian jays, ascertaining the assumptions and quantifying the relevant parameters underlying these non-mutually exclusive hypotheses is difficult.

### Evidence for selective sweeps on the Z-chromosome

We identified evidence for selection on the Z-chromosomal (negative outliers) and autosomal (positive outliers) markers with two different methods. In general, the relative frequency of outliers was higher in the Z-chromosome (67%, i.e. 4 out of 6) than in the autosomes (3%, 3/101 from the FDIST2 method and 4%, 4/101 from the BAYESFST method). Several studies of avian genomes have indeed revealed differences between the Z-chromosome and the autosomes in the rates of gene divergence (e.g. [30]), patterns of gene expression (e.g. [31]) and rates of gene movement between chromosomes (e.g. [20,22,32]), which may explain the different patterns of selection observed here. This observation is also compatible with theoretical expectation (see [33,34]) and genomic evidence (e.g. [1]) for differential selection on autosomal and Z-chromosomal (or X-chromosomal) loci: the Z-chromosome is predicted to experience selective sweeps more often than autosomal chromosomes (see [1]). Given the high levels and extent of LD observed in the population in general [21], the observed pattern of a few positive autosomal outliers out of more than 100 autosomal loci seems more consistent with locus-specific effects due to local adaptation, rather than selective sweeps. It is also possible that false positives (type I error) resulting from some statistical stochasticity or bias are responsible for the observed autosomal outliers (see e.g. [35-38]). However, the greater proportion of Z-chromosomal negative outliers may be affected by chromosome-specific effects due to sex-related evolutionary forces, which would have affected the genome in a more extensive manner. Of the loci detected to be subject to selection, three have been mapped to the chicken chromosomes (SJ009 on GgaZ, SJ022 on Gga1 and SJ036 on Gga 2; see [20]). Therefore, the loci provide good candidates for further investigations aiming to identify genes under sex-specific selection.

There are a number of sex-biased evolutionary forces (e.g. sex-biased demographic processes and sexual difference in reproductive success, lifespan and mortality rates) acting within wild birds that are known to have differential effects on loci with different modes of inheritance (see [34]). In birds, females are the heterogametic sex (ZW) whereas males are homogametic (ZZ). Because deleterious mutations will be exposed to selection on the Z-chromosome when being hemizygous in females, selection, in particular selective sweeps, may therefore favour reduced variation on the Z-chromosome leading to a balanced polymorphism [30]. In the chicken, several lines of evidence suggest that selective sweeps could be a potent force in shaping Z-chromosome variability (e.g. [23]). Another potential explanation for the balancing selection between sexes on the Z-chromosomal loci is a form of frequency-dependence selection. The selection on the sex ratio maintains males and females in the Siberian jay population with a Z/W sex chromosome system, which behaves like a single gene, as large parts of the chromosomes, including the regions containing the sex-determining region, do not undergo genetic crossing-over [39]. Since long-term balancing selection is unusual even in the classic case of *Drosophila *polymorphism [40] and the evolutionary forces are believed to be short-term here, the balancing selection on Z-chromosomal loci detected in this study should represent recent events. Balancing selection may thus often occur, although the difficulties exist in detecting the 'real' signature due to artefacts in the statistical methods (see e.g. [36]), and could be the basis for much quantitative variability, including variation in fitness [39].

Deviations from neutrality at the four Z-chromosomal loci with both the neutrality tests might have been influenced by genetic hitchhiking due to selection acting at distinct but closely linked loci. Significant evidence has been found for the balancing selection on major histocompatibility complex (MHC) genes in vertebrates including birds (see [41,42]). However, the linkage of these candidate loci to any functional loci is currently unknown. Therefore, although signatures of selection can be identified with the aid of microsatellites, microsatellites in general do not lend themselves well to studying the effects of natural selection. There are now many more tests for selection for coding and noncoding DNA sequences which need to be first identified (see [39,43]). Thus, analyses of DNA sequences have the promise to advance understanding of the different forms of balancing selection [37].

Mapping locations of the four Z-chromosomal loci (SJ009, SJ046, SJ048 and SJ108) indicated to be under balancing selection are situated in the middle of the chromosome, rather than the telomeric regions [20]. This aligns with earlier findings: selection is typically detected to act on markers in the center of Z-chromosomes in birds, as opposed to the telomeric regions (see [44]). This could be due to the generally higher recombination (and mutation) rate between loci in the centromeric than in telomeric regions along the Z-chromosome (see [20,22,45]). Alternatively, it could be also due to the fact that the pseudoautosomal region (PAR), which is hypothesized to share the properties of autosomes, is situated at one tip of the Z-chromosome in spite of its varying sizes among species. Thus, the two candidate loci (SJ009 and SJ048) in the PAR were expected to be more similar in diversity and dynamics to autosomal loci than to Z-specific loci. We speculate that the selection pattern of candidate loci in the PAR detected here may be influenced by linkage to Z-specific loci (e.g. the LD blocks in both the sexes, see Figure 5), or for some reason experiences selection intrinsic to genes surrounding them in the region. Similar evidence of a departure from a standard neutral model in PAR loci was also detected on the Z-chromosome of the Emu *Dromainus novaehollandiae *[46].

### Sex-specific patterns of LD and prospects for LD mapping within sex

Considerable amounts of LD between loci were detected within each sex. Although many forces can lead to the high levels and extent of LD, this finding as well as the long-distance LD can be potentially affected by the pedigree samples where many are full-sibs or half-sibs even within the same sex. However, since high levels of LD were still observed in the founders of the pedigree samples [21], indicating the inference is likely to apply to the whole population too. A small effective population size and closely related individuals may lie behind the high LD observed in this study, which, nevertheless, do not necessarily affect much on the comparison of LD between the sexes. We observed significantly lower LD in females than males in terms of mean *D*' values for all the synetnic marker pairs and for marker pairs separated < 10 cM. Female-biased dispersal, which may have resulted in the lower average pairwise relatedness and the lower inbreeding rate in females (see above), can provide an explanation for this. Moreover, the significantly higher genetic structuring of male population can also explain the higher LD in males.

We observed a higher level of LD for markers on the Z-chromosome than autosomes in both males and females, indicating highly variable pattern and extent of LD across the genome. Similar finding of higher LD for markers on the X-chromosome has been typically found in the X-Y sex determination system e.g. in humans (e.g. [47]) and cattle (e.g. [48]). In addition to distinct selective or mutational forces, the higher level of LD on the Z-chromosome is primarily thought to result from higher genetic drift because of its smaller effective size (3/4 of autosomes) as compared to autosomes. The fact that the increased levels of LD were also observed for markers located > 10 cM apart suggests that the factors are still operating or have been operating until recently (see [48]). Since recombination occurring between two sites will usually reduce the LD between them, the recombination rate is likely to be negatively correlated with the LD between the pair of sites. Contrary to this expectation, in general lower recombination rate but lower LD was observed in females than in males in this study. The significantly lower levels of LD on the Z-chromosome in females is mainly ascribed to the three sex-specific markers, which show relatively low LD but no recombination detected between the marker pairs (see Figure 5).

The decay of LD in both sexes of the Siberian jay population is very useful for high-resolution mapping in sex-specific association studies, provided that appropriate candidate genes are chosen. However, as observed in the samples including both sexes [21], considerable, although relatively lower levels of LD were still observed between distantly linked markers as well as between many nonsyntenic markers within the sexes. The common occurrence of nonsyntenic LD evokes serious concerns about the generation of type I errors when using LD mapping as the only means to locate genes underlying sexually dimorphic traits in the population [49]. For this purpose, a new strategy of joint linkage and LD mapping in natural populations [50] by use of an even higher density marker screening would be needed to avoid false positive results when mapping sex-specific or sex-biased genes in the population. Previous studies reported that the Z-chromosome harbours many QTLs and genes affecting traits of ecological and evolutionary importance in birds. Coupled with the LD blocks found, the higher level and decay of LD observed on the Z-chromosome in each sex suggests that LD mapping may be possible on the Z-chromosome in the bird population using low-density marker maps. In particular, the sex-specific LD mapping has the potential to be more effective in females due to the much more rapid decay of LD with distance.

## Conclusions

To our knowledge this is the first study attempting to disentangle the sexual difference in genome-wide genetic architecture in a wild bird population. We observed a difference in genetic variability, natural selection and LD between males and females in a Siberian jay population. Several different evolutionary forces and demographic processes including differential dispersal rate, recombination heterogeneity and Z-chromosomal genomic structure may underlie the observed genetic differences among sexes. Our results suggest that the sex-specific LD mapping could be promising in this population and it would be advantageous to include Z-chromosome markers. Future theoretical work (e.g. examining the joint effects of multiple evolutionary processes on genomic difference between sexes) and experimental research [e.g. aimed at sex-specific mapping Z-chromosomal QTLs or genes and investigating the evolutionary pattern of differences in gene expression (see the review by [31]) between the sexes in a wild bird population] would increase our understanding of the genetic basis of sexually dimorphic traits.

## Methods

### Study species, study population and pedigree

The Siberian jay (*Perisoreus infaustus*) is a medium sized (body mass *ca*. 85-90 g) and relatively long-lived (average generation time *ca*. 4 years) oscine passerine bird from the Corvidae family. It has a stable socially monogamous breeding system in which life-long pair-bonds are formed in permanent territories established in the coniferous forest of the northern Eurasian taiga (see e.g. [16]). The species has been described in more detail in [19,20,51].

A long-term field study of the Siberian jay (*Perisoreus infaustus*) population in Suupohja (*ca*. 66°18'N, 29°29'E) in Western Finland has been conducted since 1974. The study population, field methods and the data structure are described in detail elsewhere [20,52]. The pedigree used in this study was built based on field observations [52] and molecular tools as described in [20]. The pedigree consists of 349 animals (172 males and 177 females) sampled in 1975-2006.

### Microsatellite markers and genotypes

The pedigree has been genotyped in a total of 107 microsatellites comprising 101 autosomal and 6 Z-chromosomal markers separated by a distance of 10.6 to 229.2 cM in females and 7.7 to 168.3 cM in males [20]. A genetic map has been constructed by following the co-segregation within the pedigree and determining marker order and distances with the software CRIMAP [53], described in detail in [20]. The microsatellites were distributed in 9 autosomal linkage groups (LG1 - LG9) and one Z-chromosome linkage group (LGZ) covering in total 999 cM in females and 822.9 cM in males. A total of 938 autosomal and 15 Z-chromosomal syntenic marker pairs were obtained. The marker order and all pairwise inter-marker genetic distances are based on the sex-specific linkage maps (see [20]).

### Microsatellite variation, genetic differentiation and relatedness analyses

Microsatellite (autosomal and Z-chromosomal) variation was assessed by the number of alleles and the observed heterozygosity in the males and females using the Excel Microsatellite Toolkit version 3.1.1 [54]. Genetic differentiation between sexes was quantified by the *θ *estimator of *F*_ST _[55]. We also calculated the value of *f*, which corresponds to Wright's [56] within-population inbreeding coefficient *F*_IS_, across the loci over the sexes as well as for the total sample. These calculations were performed with FSTAT version 2.9.3.2 [57] and the significance and the 95% confidence intervals of *θ *and *f *were determined by 10 000 permutations. In addition, an index of relatedness (*R*) based on all the 107 selected loci was computed within sexes using the program RELATEDNESS version 5.08 [58]. Average relatedness was calculated across the pairwise values of males, females and all individuals, respectively. The standard deviation and 95% confidence intervals of relatedness values were estimated by jackknifing.

### Linkage disequilibrium tests

We measured the strength of LD using the Lewontin's normalised *D*' [59] modified for multiple alleles [60]. This statistic has been used earlier to measure the extent of LD between markers spanning one single chromosome and/or the whole genome in humans (e.g. [61]), livestock (e.g. [49]) and wild vertebrates (e.g. [21,32,62]). Apart from facilitating the comparison of the results with those other studies, *D*' is a convenient measure of LD as it allows use of highly polymorphic markers and is less sensitive to variation in marker allele frequencies than other measures of LD [58]. The calculations of *D*' are detailed in [34].

MIDAS (Multi-allelic Interallelic Disequilibrium Analysis Software) software [63] provided estimates of *D*'_*ij*_, *p*_i_, and *q*_i _for allelic combinations; *D*' for each marker pair were then calculated using the equations (1) in [21]. The LD was computed between all syntenic markers among the linkage groups in females and males, separately. The statistical significance (*P*-value) of the observed association between locus pairs was carried out using a Monte-Carlo approximation of Fisher's exact test as implemented in the software ARLEQUIN [64]. For each locus pair within the sex, the unbiased estimate of the *P*-value is calculated as the sum of the probabilities of all tables (with the same marginal values as the observed one) with a lower or equal probability than the observed table. Graphic summary of the extent and significance of LD determinations was displayed by using the program HaploView version 4.0 [65]. Plots of pairwise comparisons among associations relative to genetic distances were generated in autosomal and Z-chromosomal markers using Microsoft Excel.

### Tests to detect loci under selection between sexes

We examined possible departures from the standard neutral model of molecular evolution - potentially revealing demographic events or the existence of selective effects at certain loci - between sexes by the Beaumont and Nichols's modified frequentist method [66], as well as a more robust Bayesian test [67].

We used the frequentist method proposed by Beaumont and Nichols [66], further developed by Beaumont and Balding [67], and implemented in the program FDIST2, a current distributed version of the original program FDIST as described in [67]. FDIST2 calculates *θ*, Weir & Cockerham's [55] estimator of diversity for each locus in the sample. Coalescent simulations are then performed to generate data sets with a distribution of *θ *centred on the empirical estimates. We then determined the quantiles of the simulated *F*_ST _within which the observed *F*_ST_'s fell, and significance level (*P*-values) for each locus. Initially, we used an island model of population differentiation and repeated the procedure 50,000 times to generate 95% confidence intervals for neutral differentiation. Simulation parameters were under an infinite allele mutation model for 100 demes, sample sizes 100, and a mean weighted *F*_ST _of 0.0014 calculated from the 107 microsatellite loci. This method provides evidence for selection by looking for outliers with higher/lower observed *F*_ST _-values, controlling for heterozygosity [67]. This approach is fairly robust to variation in mutation rate between loci, to sample size, and whether populations are equilibrium or non-equilibrium [67].

Beaumont and Balding's [67] hierarchical-Bayesian method was performed using the program BAYESFST package [68], which generates 2000 Markov chain Monte Carlo (MCMC) simulated loci on the basis of the distribution of *F*_ST _given the data. The method combines information over loci and population in order to simultaneously estimate *F*_ST _across the locus/population combinations by a hierarchical model as detailed in [67]. Outliers from our data set were identified on the basis of the distribution following [67]. Rather assuming a fixed *F*_ST _as in the above introduced method of Beaumont and Nichols [66], this BAYESFST test uses more information from the raw data and does not assume the same *F*_ST _for each population [67,69].

## Authors' contributions

MHL designed the study, performed the data analysis and wrote the manuscript. JM planned and coordinated the whole study, and contributed to the manuscript writing. Both authors read and approved the final manuscript.
